# Gene make-up: rapid and massive intron gains after horizontal transfer of a bacterial α-amylase gene to Basidiomycetes

**DOI:** 10.1186/1471-2148-13-40

**Published:** 2013-02-13

**Authors:** Jean-Luc Da Lage, Manfred Binder, Aurélie Hua-Van, Štefan Janeček, Didier Casane

**Affiliations:** 1 Laboratoire Evolution, génomes et spéciation UPR 9034 CNRS, 91198 Gif-sur-Yvette, and Université Paris-Sud, Orsay, 91405, France; 2 CBS Fungal Biodiversity Centre, Evolutionary Phytopathology, Institute of the Royal Netherlands Academy of Arts and Sciences (KNAW), Uppsalalaan 8, Utrecht, CT, 3584, The Netherlands; 3 Laboratory of Protein Evolution, Institute of Molecular Biology, Slovak Academy of Science, Dubravska cesta 21, Bratislava, SK-84551, Slovakia; 4 Université Paris Diderot, Sorbonne Paris Cité, France

**Keywords:** Glycosyl hydrolase, Lateral gene transfer, Fungi, Gene duplication, Intron gain, Protosplice

## Abstract

**Background:**

Increasing genome data show that introns, a hallmark of eukaryotes, already existed at a high density in the last common ancestor of extant eukaryotes. However, intron content is highly variable among species. The tempo of intron gains and losses has been irregular and several factors may explain why some genomes are intron-poor whereas other are intron-rich.

**Results:**

We studied the dynamics of intron gains and losses in an α-amylase gene, whose product breaks down starch and other polysaccharides. It was transferred from an Actinobacterium to an ancestor of Agaricomycotina. This gene underwent further duplications in several species. The results indicate a high rate of intron insertions soon after the gene settled in the fungal genome. A number of these oldest introns, regularly scattered along the gene, remained conserved. Subsequent gains and losses were lineage dependent, with a majority of losses. Moreover, a few species exhibited a high number of both specific intron gains and losses in recent periods. There was little sequence conservation around insertion sites, then probably little information for splicing, whereas splicing sites, inside introns, showed typical and conserved patterns. There was little variation of intron size.

**Conclusions:**

Since most Basidiomycetes have intron-rich genomes and this richness was ancestral in Fungi, long before the transfer event, we suggest that the new gene was shaped to comply with requirements of the splicing machinery, such as short exon and intron sizes, in order to be correctly processed.

## Background

The ongoing debate on the origin and evolution of spliceosomal introns in eukaryotes has shifted in the last few years on the origin of variations in intron density in genomes, and correlatively, on the relative rates of gain and loss of introns. Indeed, whole genome sequencing of a variety of eukaryote species has revealed an impressive diversity of intron contents. There are intron-poor species, mostly unicellular, such as *Saccharomyces cerevisiae*, *Guillardia theta*, *Encephalitozoon cuniculi*. Intron-rich species are often multicellular, for example vertebrates, the worm *Caenorhabditis elegans*, the fungus *Phanerochaete chrysosporium*, the sea squirt *Ciona intestinalis*. Intron-rich unicellular organisms also exist, like the green alga *Chlamydomonas*[1,2]. Several studies have concluded that the last eukaryote common ancestor (LECA) had a mild to high intron density (e.g. [1,3-7]. However, it seems that the subsequent history, during lineages diversification, has been quite diverse, with massive losses in some lineages, bursts of intron gains followed by either stases or losses, or reset of intron positions in others [1,2,4,7-12]. Several possible reasons have been proposed to explain the contrasted current situation: low population sizes allowing fixation of mildly deleterious introns [13], variable balance between different mechanisms of DNA repair [14], selection for optimal exon size, due to spliceosome requiremements [15], nonsense mediated decay (NMD) [16]. Many studies have shown a large excess of intron losses relative to gains, especially when related species were compared [9,17-22]. Comparisons of a single gene among more or less related species have also suggested that intron losses outnumbered intron gains since the split of the species studied from their common ancestor. Moreover, repeated, independent loss of the same intron (in the same position) was often noticed [23-27]). In contrast, until recently, clear recent intron gains had not been frequently identified (e.g. [28,29]). Some gain cases inferred from a given data set appeared, after further sampling, to be recurrent losses [22]. However, some cases of gains outnumbering losses were reported in fungi [30]. Indeed, recent population genomic studies and increasing sequence data show that gains are still occurring [31,32]. We still have little knowledge of the real tempo of intron gains and losses during evolution along a lineage and the factors that influence it. Dynamics of intron gains and losses in the course of evolution is an attracting issue, given its biological significance. A method for addressing this issue is to survey eukaryote genes horizontally transferred recently from bacteria, which are devoid of spliceosomal introns [21]. Recent transfers followed by intron insertions may give insights into the pace and dynamics of gains, provided that the HGT could be dated.

In vertebrates and possibly other intron-rich genomes, it has been shown that exons exceeding a certain size may be misrecognized by the splicing machinery [15,33], or prone to premature termination codons, due to the unability for NMD to act upon [16]. We hypothesize that in such intron-rich genomes, intronless genes stemming from horizontal transfer from bacteria should be quickly invaded by introns to shorten the exon size. The NMD hypothesis also posits that introns should be inserted regularly along the gene. Indeed, a study of HGT genes in fungal genomes, mostly Ascomycetes, showed a correlation between intron densities in transgenes of bacterial origin and the recipient genomes [34]. Here we studied an α-amylase gene, previously identified in a Basidiomycete, the white rot *Phanerochaete chrysosporium*[35], that was transferred from an Actinobacterium to Agaricomycotina. Alpha-amylases often form multigene families, and most Basidiomycetes already harbor at least one fungal-type α-amylase gene (Carbohydrate Active Enzymes database http://www.cazy.org[36]). Basidiomycetes are ancestrally intron-rich [7]. In this new gene of bacterial origin, we have identified intron gains and losses that occurred since the gene settled in the fungal genome and we estimated the rates of gains and losses, and some characteristics of the introns inserted.

## Methods

The sequence jgi|Phchr1|7087| from *Phanerochaete chrysosporium*, already reported to encode an animal-type α-amylase [35] was used as a query for BLASTP search in GenBank nr and GenBank Fungal Genomes (http://blast.ncbi.nlm.nih.gov), and BLASTP search implemented in the Mycocosm data base at the Joint Genome Institute (http://genome.jgi-psf.org/programs/fungi/, [37]). The putative retrieved orthologs were then aligned using MAFFT [38] implemented in the Geneious software (Biomatters Ltd.), and manually corrected for erroneous intron-exon structures when necessary. Those errors were detected when large unique amino acid insertions or deletions were evidenced in the alignment. In these cases, when available and if necessary, expressed sequence tags (EST) were used to confirm intron positions and boundaries. The query sequence contained a C-terminal carbohydrate binding module of the CBM20 family. A number, but not all retrieved sequences possessed a terminal CBM20 domain of variable length, always containing introns. Because it was not present in every sequence, the CBM20 was no longer considered and the alignment was truncated to the C-terminal end of the core protein. Intron positions were mapped onto the alignment according to the annotations of the genomes, mainly those deciphered at the JGI. From this protein alignment, after curation of the alignment with Gblocks [39] leaving 398 positions (83%) available, a gene tree was built using PhyML [40], at the http://www.phylogeny.fr web server [41]. After testing various models with MEGA5 [42], we used the WAG substitution matrix with a gamma distribution of substitution rate across sites (the shape parameter α was estimated from the data with four rate categories). The robustness of the nodes was estimated by 100 bootstrap replicates.

A few species were also investigated experimentally using polymerase chain reaction. DNA samples were supplied by the Hibbett Laboratory at Clark University or purchased from the Centraalbureau voor Schimmelcultures at the Institute of the Royal Netherlands Academy of Arts and Sciences. The primers and experimental conditions are given in Additional file 1: Table S1. Only partial sequence data were obtained from the following species related to *P. chrysosporium*: *Phlebia radiata* FPL6140*, P. albomellea* CBS 275.92*, Grifola frondosa* MO11 (accession numbers JX310736-JX310738).

In order to infer the antiquity of the α-amylase gene transfer from a bacterium, and the times of intron insertions, we estimated the ages of nodes in a species tree. A fungal species tree was established by compilation of recent literature, which included the species of interest for our study, but also Ascomycetes ([43-51] and especially [52]), and unpublished data kindly shared by D. S. Hibbett and by the Joint Genome Institute (Binder et al. in preparation) for solving uncertain relationships. We performed a Bayesian analysis with the BEAST program [53]. An alignment was performed for 54 fungal species, using protein sequences of elongation factor 1-alpha, RNA polymerase II largest and second large subunits (EF1α, LSU1 and LSU2, Additional file 2: Table S2) aligned separately using MAFFT [38], then concatenated. After curation for badly aligned regions with Gblocks [39], 1671 amino acid positions remained. The tree made from the alignment was constrained to match the established species tree topology. We estimated divergence times using BEAST v1.7.1 [53], assuming a relaxed uncorrelated lognormal molecular clock model, a Yule speciation process for tree prior, and a WAG + Γ substitution model. The analysis was run for 12 million generations, saving a tree every 1,000^th^ generation. The resulting log file was inspected with Tracer v1.5 [54] to verify that the sample size was large enough to give good estimations of posterior distributions. We found that the steady state had been already reached after two millions generations. After removing the first 2,000 trees as burn-in, the remaining 10,000 sampled trees were analyzed with TreeAnnotator v1.7.1 [53] to estimate the 95% highest posterior intervals of the divergence times. Fossil calibration was possible at two nodes : divergence between Ascomycetes and Basidiomycetes was set to 600 Ma [55], and divergence between Eurotiomycetes and Sordariomycetes was set to 410 Ma, the age of the oldest likely Sordariomycete [56,57].

In order to show the occurrence of HGT and its origin, a general gene tree of glycosyl hydrolases of the GH13 family, which have a broad activity range [58], from various organisms was built from a structural alignment as described in ref. [59], adding the sequences studied here.

Gains and losses of introns were inferred in a weighted (Dollo) parsimony framework, considering parallel losses much more frequent than parallel gains [60], as in [27]. Using Mesquite v. 2.75 [61], we tried parsimony and ML scenarii for intron gains and losses directly on the gene tree. Because numerous gene duplications and gene losses occurred, we tried to reconcile the gene and species trees using Notung 2.6 [62]. The program MALIN [63] infers the evolution of exon-intron structure in protein-coding orthologs. It could not be used, though, because orthology relationships among the genes could not be solved in most cases (see Results). Finally, the loss and gain events were mapped onto the species tree, not the gene tree. The average rates of intron gains and losses per million year and per branch were computed by considering that events occurred evenly along a branch. For example, if three losses occurred along a branch 12 Ma in length, the loss rate was 3/12 per Ma. Then, the rates for all branches present at a given time were summed and averaged.

## Results

### Gene transfer from a bacterium

**Figure 1:**
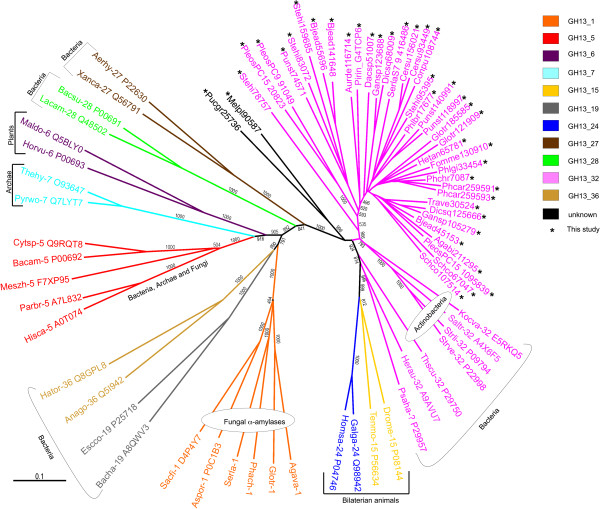
**Tree of glycosyl hydrolases of the GH13 family.** Full gene and species names and taxonomic positions are given in Additional file 3: Table S3. GH13 subfamilies [58] are colour-labelled and indicated by their numbers next to the species names. The fungal genes studied in this work are indicated by an asterisk. Bootstrap values are shown along the branches.

We first performed TBLASTN and BLASTP searches against GenBank using the candidate α-amylase gene Phchr1|7087|. The best hits belonged to a few Basidiomycetes (*Serpula lacrymans, Schizophyllum commune, Piriformospora indica*) and then a lot of Bacteria, mainly Actinomycetales. No other fungus was found within the 100 first hits, except *Moniliophthora perniciosa* (Agaricales, Marasmiaceae), a truncated sequence which will be no longer considered here (MPER_11606), and a single Ascomycete species, *Chaetomium globosum*, already reported to harbor a similar α-amylase gene (CHGG_04966), but with a distinct bacterial origin [35]. However, most fungal genome data have not been deposited yet to GenBank. Thus, we searched for genes similar to our *P. chrysosporium* query in the Mycocosm database at the JGI. Our BLAST searches against all fungal database available to us (fungalgenomes.org) retrieved a total of 42 sequences with high similarity to the *P. chrysosporium* query (BLASTP expect-value < 10^-109^ in the Mycocosm database) from 24 species only, all Basidiomycetes. This confirmed the limited phylogenetic distribution of this gene among Fungi, and thus supported its likely bacterial origin. Figure 1 shows a tree of α-amylases of the GH13 family [64] from various prokaryotes and eukaryotes. This important enzyme group was divided in subfamilies [58]. The tree shows that the genes we recovered in Basidiomycetes are grouped among Actinobacteria GH13_32 α-amylases, supporting an actinobacterial origin of the donor species. A very recent study supports this conclusion [65]. The phylogenetic distribution of the recovered genes is limited to Agaricomycotina, suggests that the HGT event took place rather basally in Basidiomycetes, but after the split from Tremellomycetes, probably at the basal node of Agaricomycotina. Interestingly, according to the phylogenetic distribution of the genes, a few species seem to have lost this α-amylase: the clade containing *Postia placenta*, *Wolfiporia cocos* and *Fomitopsis pinicola,* and the clade containing *Coprinopsis cinerea* and *Laccaria bicolor*. The Bolete *Paxillus involutus* also lacks the gene (not shown). The gene was duplicated independently in several lineages, with for instance four copies in *Stereum hirsutum*. In addition to these 42 sequences, two other Basidiomycete sequences from the remote Pucciniomycetes *Melampsora laricis-populina* (Melpl1|90587|) and *Puccinia graminis* (Pucgr1|25736|) were retrieved, with much lower similarity with the *P. chrysosporium* query (expect value ca. 10^-67^ and 10^-76^, respectively), but they probably have an origin distinct from the gene studied here, although bacterial too, given their position in the tree (Figure 1 and Additional file 3: Table S3).

### Intron richness, gains and losses

**Figure 2:**
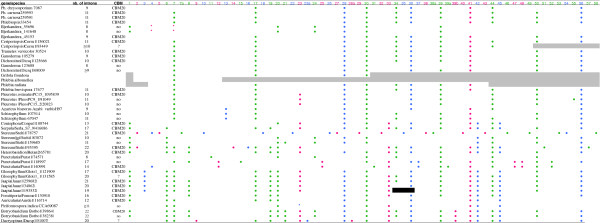
**Intron positions in the transferred α-amylase gene homologous to Phchr1|7087| found in Fungi.** Pink dots: phase 0 introns; green dots: phase 1 introns; blue dots: phase 2 introns. Shaded regions represent unknown sequences. The black bar is a region of uncertain annotation. Asterisks indicate possible cases of intron sliding. CBM20 indicates the presence of an additional carbohydrate binding module of the CBM20 family. The number of introns is meant without the CBM20 extension.

As many as 480 introns that map at 64 intron positions were identified (Figure 2 and Additional file 4: Figure S1). The number of introns per gene ranged from 8 (*Ganoderma* sp. Gansp1|123688|) to 22 (e.g. *Stereum hirsutum* Stehi1|95395|), with 13.3 introns/gene on average, not counting the CBM20 extension. This high density considerably exceeds the average values for Basidiomycete genomes, which range from 3.8 to 5.7 introns/gene (data from the JGI). In addition, we found no correlation between the average genomic intron density and the intron density in the HGT *Amy* genes (not shown), in contrast to a previous study [34]. This may be explained by the fact that all the species in our study fall within a relatively narrow range of genomic intron density, compared to the span of the study cited above, which included Ascomycetes, that are more intron-poor, and by the likely large within-genome variance. We were unable to identify the origin of any inserted introns (donor DNA). Indeed, intron sequences diverged too fast to allow alignment between orthologous introns even between closely related species, such as *P. chrysosporium* and *P. carnosa*.

**Figure 3:**
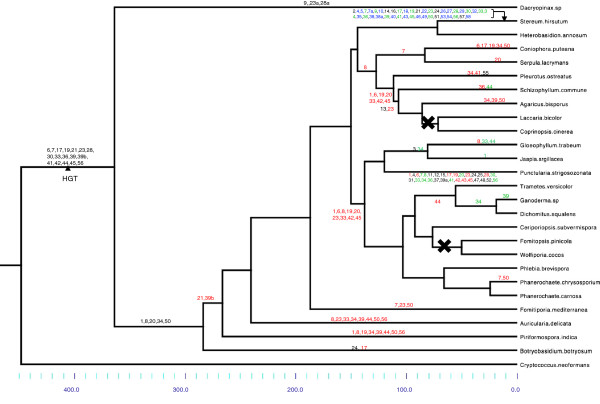
**Reconstitution of intron gain and loss events mapped on a species tree.** Branch lengths are proportional to time, the time scale in million years is shown below. Numbers along the branches are intron positions. HGT: horizontal transfer event. Black numbers: intron gains; red numbers: intron losses in all gene copies in a clade; green numbers: intron loss in some, but not all gene copies in a clade; blue numbers: intron gains specific to Stehi1|78757|. Black crosses show complete gene losses.

In order to reconstruct the history of intron insertions and losses, we ideally should map the intron gain and loss events onto the gene tree, applying a parsimonious or maximum likelihood model. We tried to apply a parsimony analysis on the unmodified gene tree using Mesquite v2.75 [61]. This led to at least 23 occurrences of parallel gains, counting parallel gains as the number of gain events at a given intron position, minus one. We obtained a similar result in a maximum likelihood analysis, parametered with a bias of gains over losses of 1:10 (not shown). Actually, the gene tree built from our data (Additional file 5: Figure S2) had a number of weakly supported nodes and was incongruent with the currently known species phylogeny, and thus failed to clarify the history of the gene family since the time it was transferred into an ancestral genome. High divergence between gene copies, multiple independent duplications and paralog losses may have obscured the phylogenetic signal. HGT may also have occurred between fungal species (see e.g. ref. [66]). However, our data show no clear evidence for this, except for *Bjerkandera adusta* (see below). Studying synteny among species (Mycocosm website) was no more helpful to uncover orthology relationships, because of quick loss of synteny, except in closely related species. Therefore, we attempted to reconcile the gene tree with the known phylogeny using the Notung software [62]. We obtained a complex history, with 19 duplications and 56 gene losses (default parameters, with rearrangement option and rooting with Stehi1|78757|, Additional file 6: Figure S3). Moreover, some major branches were marked as weak by the program. This may be due to the low support values at a majority of nodes (Additional file 5: Figure S2). Therefore, we mapped the intron gains and losses on a species tree (Figure 3) from the data of Figure 2, in a weighted parsimony framework. With this method, the possible parallel gains were limited to positions 2, 4, 21 and 24. Intron gains were rather easily inferred. Clearly, there has been a relatively rapid invasion of the primarily intronless gene by introns after its transfer into the ancestral genome. According to our reconstruction, 17 extant introns are ancestral, since they are still shared together by the single copy of the early branching-off *Dacryopinax sp.* and a number of other species. Among them, 9 are still widely distributed. Note that *Dacryopinax sp.* has three specific introns. We considered those introns as specific gains, but this cannot be ascertained without additional data from other early diverging species. To infer intron losses, when several copies were present in a species, for each intron position, we distinguished between intron losses in all gene copies, and intron losses in some, but not all gene copies. Intron losses in all copies were counted as a single event, because it was rarely possible to discriminate between parallel losses in paralogs and a single event prior to duplication, thus probably underestimating the rate of loss. Some examples for which the gene tree was clear enough to allow more precise reconstitution of the loss events, were e.g. partial losses of introns 34 and 39 in *Ganoderma* sp. and its relative *Dichomitus squalens*, or independent losses at positions 33 and 34 in *G. trabeum* Glotr1|121909| and *P. strigosozonata* Punst1|74571|. Figure 3 shows that the same set of introns (1, 6, 19, 20, 33, 42, 45) was lost twice, at two internal nodes, the node basal to Agarics and the node basal to Polypores. This intriguing result of our reconstruction might reveal hidden paralogy, but the gene tree was not clear enough to validate this possibility. Indeed, parallel losses were observed many times in this study, and are generally considered to be much more frequent than parallel gains. However, it seems unlikely that such a co-occurrence of parallel losses may have occurred by chance. On the other hand, the Notung reconciliation assay was not consistent in this respect, because it did not propose to group as orthologs the two clades that have lost this set of introns, as would be expected if we infer a single occurrence of the loss of the seven introns. Similarly, we considered that another set of introns (8, 34, 39, 44, 50, 56) was lost independently along two external branches, *Piriformospora indica* and *Auricularia delicata*. Simulation (100,000 trials) suggested that the probability of such 6 parallel losses among eight intron losses in a pool of 20 introns was about 1%, at most 3% when considering that some positions were lost more frequently (estimated by the actual rate observed in our data set). In the case of these two species, the gene tree suggested a relationship between the single-copy genes present in both species. This could represent the remnant gene copy of two ancestral copies, which was lost in the ingroup clade, whereas, on the contrary, the remaining ingroup copy would have been lost in those two species. Note that the Notung reconciliation suggests an ortholog relationship of these two genes too. Then, possible hidden paralogy leads to overestimating the rate of losses.

**Figure 4:**
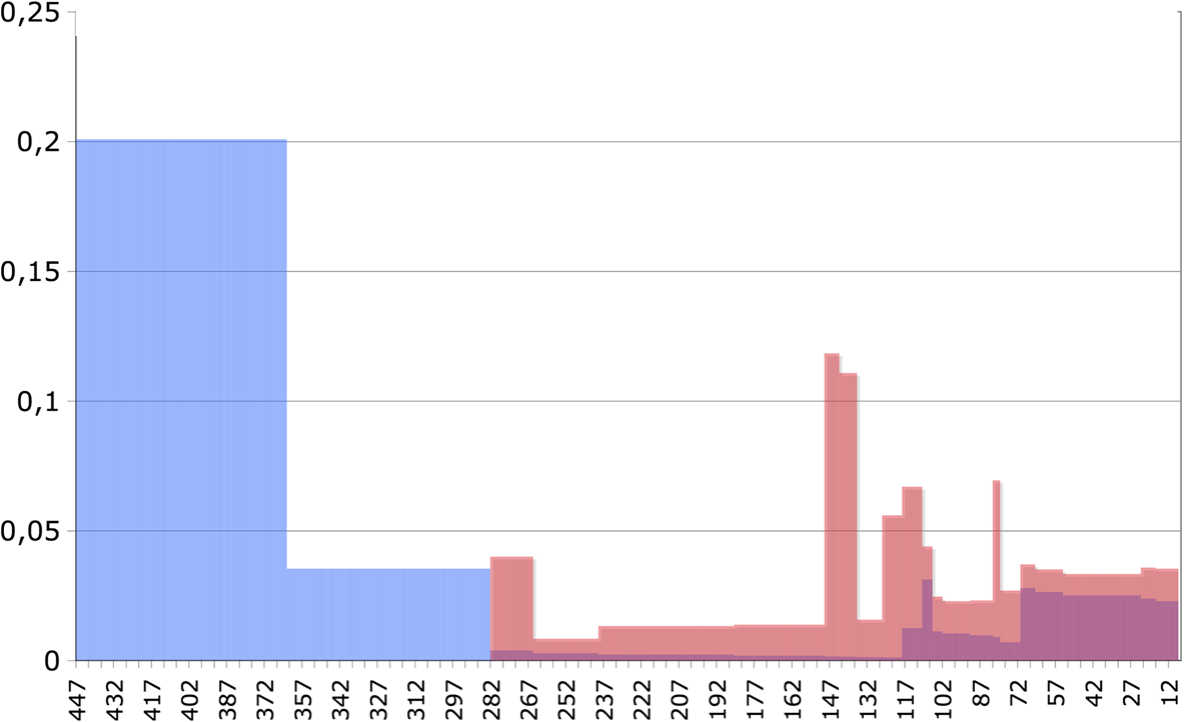
**Apparent intron gain and loss rates per million year and per lineage.** Gain rates are in blue, loss rates are in red. The X axis is graduated in million years from the present.

We attempted to date divergence times of various fungal clades in order to date intron gains or losses (Additional file 7: Figure S4). These values were flawed by a large variance, due in part to the scarcity of fossils to be used for calibration and their datation. We estimated that the gene transfer occurred 448–363 million years ago (Ma). This means that at least 17 gains occurred within a ca. 85 Ma period, which is a high rate for a single gene. The average *apparent* rates of gains and losses per million year and per lineage are shown on Figure 4. This graph shows that after the initial burst of gains, few gains took place whereas losses accumulated. There were exceptions in two terminal branches, *Stereum hirsutum* and *Punctularia strigosozonata*, which both experienced numerous specific gains and losses. These two species are the main contributors to the second rise of gains in recent times in Figure 4.

### Intron sizes and insertion sites

**Figure 5:**
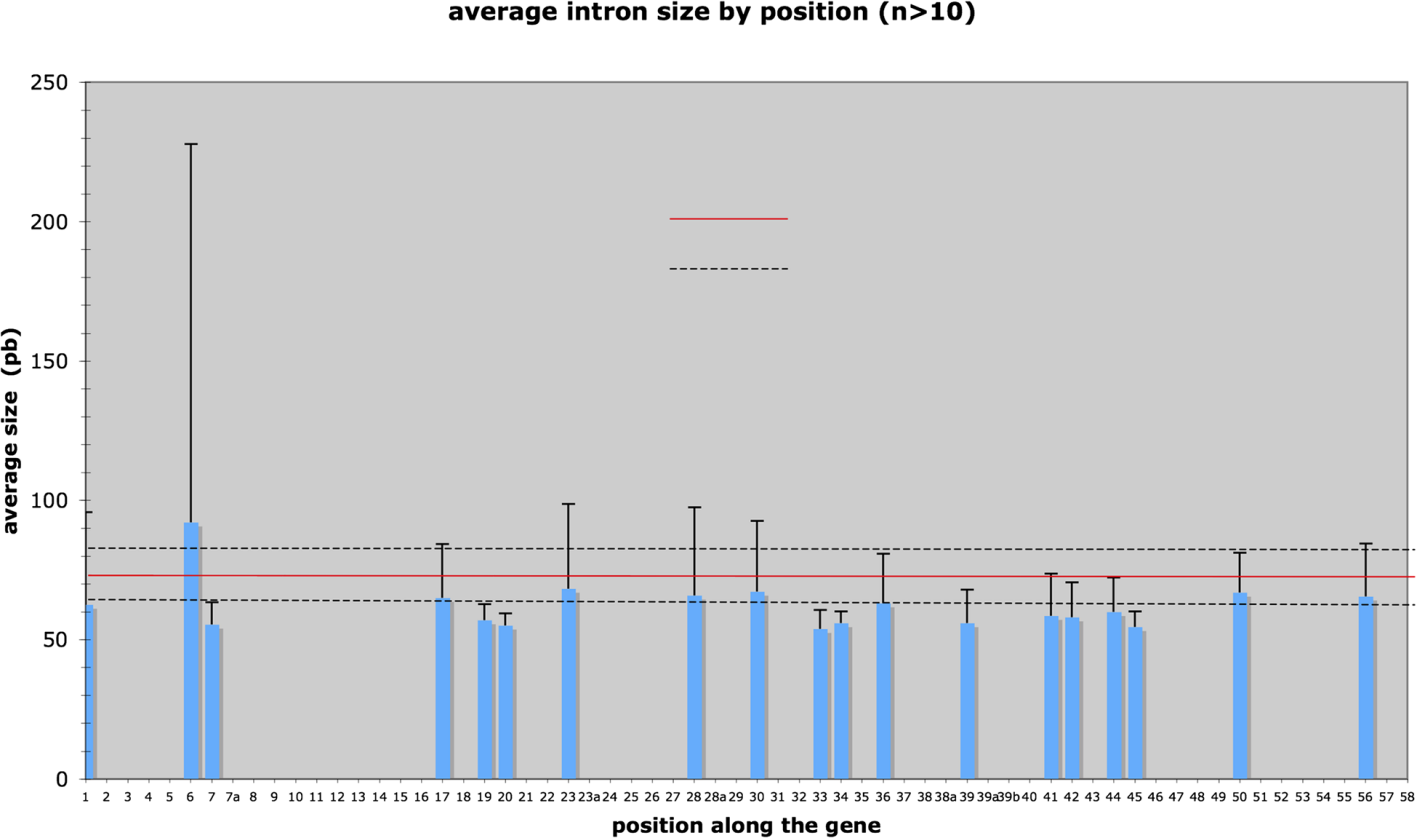
**Average intron sizes at positions where more than ten values were available.** Error bars indicate standard deviations. The long bar at position 6 is due to a single long intron in Stehi1|83072|. Red line : average intron size for the whole genomes of all the species studied. Dashed lines show standard deviation.

Overall, the average intron size was 61.6 bp, with a low dispersion, since the median was 56 bp and the third quartile was 62 bp. Figure 5 shows the average intron sizes at positions with more than ten values available. The sizes fall well within the range of average intron sizes at the genome level for the species included in the study. The conspicuous size homogeneity across intron positions and the generally low standard deviations suggest that intron size, at least in this "young" gene, may be constrained, e.g. to fit the abilities of the spliceosome.

In eukaryote genomes, an excess of phase 0 introns was often observed [67,68], including in Fungi [30]. We did not find such a bias, but rather a slight excess of phase 1 introns (28/64), however not significantly different from a 1:1:1 distribution (χ^2^ = 3.28 n.s.), not counting the putative slided introns. Results were similar for the 17 oldest intron positions.

We noticed no spatial preference for intron insertion (homogeneity test ; the alignment was divided in ten parts of equal length, p = 0.71, n.s.), except that there were no introns in the putative signal peptides. The NMD hypothesis suggests that introns colonizing an empty gene would be prone to regular spacing [16]. We checked whether the 17 ancestral introns were inserted at random or showed a regular pattern along the coding sequence. There was no over-regularity compared to random spacing for these oldest intron positions, as estimated by simulating 10,000 genes with 17 random insertions (p = 0.16). All the extant genes from our data set were checked as well. Overall, there may be some trend towards regular spacing of introns, since about half of the genes showed intron spacing significantly more regular than expected by chance (Additional file 8: Table S4).

**Figure 6:**
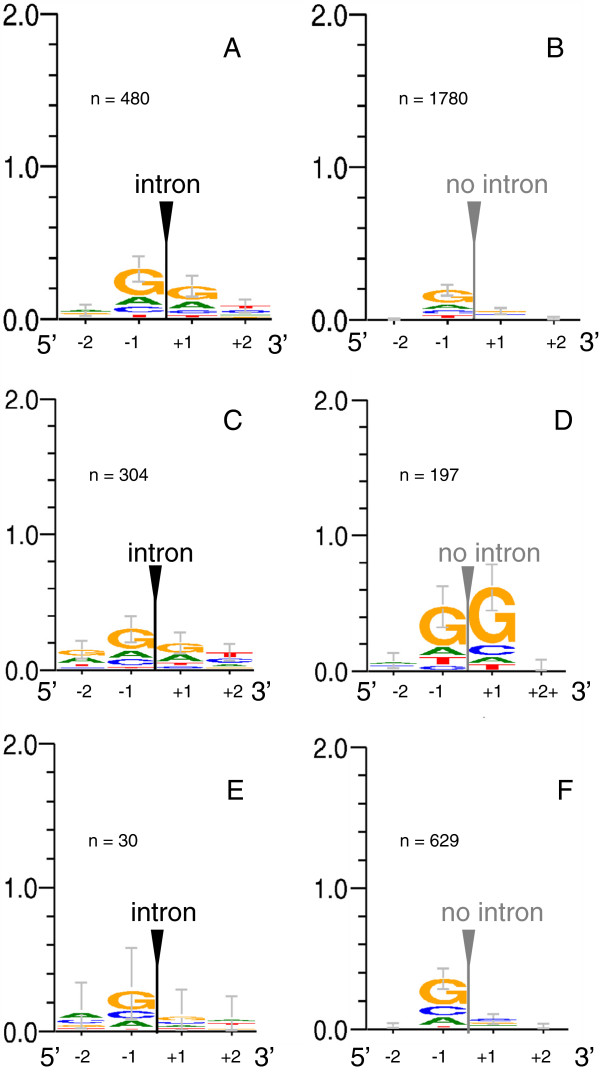
**Consensus sequences at positions −2 and −1, and +1 and +2 around intron positions, drawn with Weblogo 3.2**[72]**(**http://weblogo.threeplusone.com/create.cgi**)**. **A**: all positions, intron present. **B**: all positions, intron absent. **C**: sequence at the 17 oldest positions when an intron is present; **D**: sequence at the 17 oldest positions in the absence of intron, i. e. after intron loss; **E**: sequence at the recent positions 2, 3, 4, 11, 12, 13, 14, 15, 16, 21, 25, 31, 37, 39a, 47, 48, 51, 52, 55, 57 in the presence of intron; **F**: same positions as E, in the absence of intron. Introns of Stehi_78757, of *Jaapia argillacea* and of *Bjerkandera adusta* were not included. n is the number of sequences. Y-axis is graduated in bits of information. Error bars are Bayesian 95% confidence intervals.

We studied whether introns were inserted into preferential sequences, according to the protosplice model [69], and whether local sequence information changed in the presence vs. in the absence of introns. The major fact is that overall, considering all intron positions and phases, there was little information at the last two exonic 5' and the first two exonic 3' nucleotide positions (Figure 6A and 6B). A slight preference for G[intron]G was suggested, more visible for phase 1 introns (Additional file 9: Figure S5), as observed earlier (e.g.[70]). When the intron was absent, the level of information was even lower. This lower information level in the absence of intron was observed for each phase considered separately (Additional file 9: Figure S5). We also compared information level according to the age of insertions, i.e. recently inserted introns *vs.* the 17 oldest positions. Information was slightly stronger around oldest introns than around more recent introns, although not significantly (Figure 6C and 6E). Conserved oldest introns, i.e. still present in most extant genes, had not a higher informational environment (not shown). One might assume that their presence in a majority of genes until now might be related to a strong splicing signal, avoiding missplicing, and thus negative selection. We observed that their conservation until now could not be explained by a more informative environment. In contrast, the level of information increased at oldest positions after intron loss (Figure 6D). Information content inside the introns was investigated at the 5' and 3' splicing sites. Sequences complied to a classical consensus GTrnG…yAG. Thirteen introns over 478 (2.7%) had GC instead of GT as the donor site, consistent with the 1.3% reported by Iwata et al. (2011). There was a strongly conserved G at position 5, as noticed in Fungi [71]. However, the prevalence of this G varied according to the intron position (e.g. 30 vs. 56) or the species (Additional file 10: Figure S6). Recent introns showed a lower information content at positions 3 and 4 of the 5' splicing site; however, this may be not significant given the limited sample size.

### The case of *Bjerkandera adusta*. Intron sliding

*Bjerkandera adusta* is a close relative of *Phanerochaete chrysosporium*. It was not included in the analysis shown on Figure 3 and Figure 4 because, intriguingly, none of its three gene copies is close to the ones of *P. chrysosporium* or its other relatives. In addition, two copies (Bjead1|55696|, Bjead1|141648|) share highest sequence similarity and three intron positions (1, 20, 24) exclusively with remote species such as *Stereum hirsutum* (Russulales). This illustrates the complicated gene history and might raise the possibility of HGT among fungi. It is also worth noting two occurrences of intron sliding in those copies of *B. adusta* at positions 4 and 7 (marked by asterisks in Figure 2). Intron 4 is absent from most genes. Thus, one can hypothesize an independent gain, rather than displacement of a preexisting intron. In contrast, in the case of the widespread intron 7, although it is absent from the closest sequences Punst1|74571|, Stehi1|83072| and Stehi1|159685|, one could more likely infer an intron displacement, one base pair apart (phase 0 vs. phase 1). This new phase 0 intron is located at the same position as the widespread intron 1 of animals [27]. Intron sliding was also found in *Piriformospora indica* (ancestral position 23).

### The case of *Stereum hirsutum* Stehi1|78757|

We found four copies in *Stereum hirsutum*. Stehi1|78757| was the most diverged sequence among all our data set. Strikingly, most of its 21 intron positions were different from the positions found in the other genes (Figure 2). This pattern could be explained by an independent gene insertion from an intronless donor, such as a bacterium or a retrotranscript from an existing copy, followed by *de novo* intron colonization. Whatever the origin ot this gene copy, it highlights the high intron density in a gene of likely recent origin. Note that two possible cases of parallel intron gains at positions 2 and 4 involve positions found in this copy. This reinforces the hypothesis of true parallel gains. The specific introns of Stehi1|78757| account for one third of the whole number of intron positions.

## Discussion

For studying the dynamics of intron gains in eukaryote genes, it is worth using primitively intronless genes, originating from either bacteria or retro-elements. A few such studies were published recently [73,74]. In a previous study [27], we had investigated the dynamics of introns in the α-amylase genes of bilaterian animals, likely of bacterial origin [35]. The putative HGT event was about twice as old as the one studied here. We had retrieved at most three likely ancestral intron positions and only a minority of positions were shared by several phyla, so that it was not possible to infer the pace of intron colonization after the gene insertion. In contrast, in this study, we have shown that a gene of bacterial origin, transferred horizontally into a fungus, was quickly split by numerous introns about 300–400 million years ago. The donor was an actinobacterium, and it is likely that this kind of transfer happened several times independently. Indeed, the related α-amylase gene found in *P. graminis* and *M. laricis-populina* is most likely the result of a different transfer event. The position of these sequences in the gene tree (Additional file 5: Figure S2) is clearly not related to the sequences studied here. Moreover, the sequences of these two species share 12 intron positions, ten of which are different from the 64 positions identified in our study, and two positions are common with the "outlier" gene Stehi1|78757|. A similar situation was observed in the *nad7* gene, transferred twice independently from mitochondrion in Opisthokonts and in *Chlamydomonas reinhardtii*[73]. We have no evidence that the carbohydrate binding module CBM20 was co-transferred with the core enzyme gene. CBM20 domains exist in bacteria, but they are common in fungal glycosyl hydrolases too [75,76], e.g. in the GH15 family, and may have been recruited later in the HGT α-amylase gene through domain shuffling.

The time of the HGT is uncertain, given the scarce fossil data that can be used for time calibration. Therefore, the dates computed here are indicative. It seems however clear that at least 17 introns were inserted within a rather short period, before the divergence of *Dacryopinax sp.* The origin of these introns could obviously not be retrieved, given the long time elapsed. Even for more recent introns, e.g. specific introns found in *P. strigosozonata* or *S. hirsutum*, it was not possible to identify any donor sequence. Indeed, among the various mechanisms proposed for intron gains [2,32,77,78], it has been shown that new introns may be created by random insertion of any DNA fragment or nucleotide filling during DNA repair after double strand break [14,31,77,79], which thus form novel sequences. Comparisons between closely related species, such as *P. chrysosporium* and *P. carnosa* also shows the fast divergence of introns.

Inferring gain and loss events along lineages was made difficult by the lack of congruence between the gene tree and the species tree. Relationships between genes were not clear, bootstrap supports were often low. As mentioned above, hidden paralogy was suspected is several cases. Only substantial additional data from other species could help in solving this problem, which led to overestimate the loss rates at some periods. Gains were generally easily mapped, except a few cases where parallel gains were proposed. Parallel losses occurred much more frequently than gains, even not counting the possibly misleading hidden paralogies. Intron 7 was lost five times, intron 34 at least eight times. This is consistent with many reports showing that parallel losses are common relative to parallel intron gains (e.g. [60,80]). Correlatively, we have shown that, after the initial burst of gains in the empty gene, the rate of gains dropped and the rate of losses increased up to a large excess of loss over gain, as already observed [80,81]. The high activity of specific gains and losses in a few terminal branches of our data set, *P. strigosozonata* and *S. hirsutum*, remains unexplained, especially as there is no such activity in their relatives (*G. trabeum* and *H. annosum*, respectively). This could be related to the occurrence of several copies, four and three, respectively. High rates of intron gains and losses were reported in paralogous genes [82]. In *S. hirsutum*, a lot of specific gains occurred in a particular gene copy, Stehi1|78757|, which has probably a quite different history. It is unclear whether this copy originated in an independent HGT from a related bacterium or stems from a processed cDNA. In the latter case, there should be some sequence similarity with the parent gene, which was not found in the extant gene copies of this species. This gene must have been acquired much more recently than the gene shared by most Agaricomycotina. And yet, it is very intron-rich. This point adds relevance to our hypothesis that primitively intronless genes in intron-rich genomes are prone to be quickly provided with interrupting sequences. Rapid acquisition of introns was also observed in mitochondrial-derived genes, assumed to be primitively intronless [73] and in mammalian "domesticated genes" stemming from tranposable elements [74].

The HGT α-amylase gene could be a suitable model for studying the evolution of information content around intron sites. However, we found a low level of information at positions −2 to +2 surrounding intron sites, contrasting with our results in animals [27], where the classical AG/G protosplice consensus [69] was majoritary. This may indicate an absence of insertional sequence preference. As in our previous study, we noticed an even weaker level of information around empty sites. Information was not significantly stronger around older introns than recent introns, but this result suffers from a low number of data and a high variance for recent insertions. As underlined by Rogozin and colleagues [80], evolution towards the protosplice consensus may be a slow process, and our gene may be too recent. The increase of information after loss of old introns is surprising, because if intron neighborhood is involved in intron recognition and splicing, which is well established, one would rather expect a relaxation of constraints after intron loss, thus unbiased base composition.

In contrast, information at both 5' and 3' splicing sites was strong and typical of fungal introns [71], suggesting that, whereas exonic neighborhood may be not crucial for splicing, intronic splicing sequences are important for proper intron recognition. Another important feature for efficient splicing may be a short intron size. Indeed, we have shown the low variability of size in our data set, whatever the species and the intron position. This could be indicative of a functional constraint. This is consistent with [71], who have shown that short intron sizes contributed importantly to intron detection in Basidiomycetes.

## Conclusion

Altogether, our data suggest that several features were important to confer to the transferred gene suitable characteristics regarding splicing efficiency: short introns; shortening the exons to a small size through multiple intron gains, although exon sizes were more variable than intron sizes; and rather regular intron spacing along the entire gene, perhaps for efficient nonsense mediated decay [16]. It is not clear whether intron gains were positively selected. It has been proposed that introns colonized eukaryotic genomes by random fixation in low population size species, while they were mildly deleterious [13,83,84]. However, in our case, the HGT gene settled in a genome that was probably already intron-rich [7], endowed with a spliceosome adapted to cope with intron-rich genes. The potential deleterious effect of inserting an intron might have been balanced by the advantage of splitting the gene in smaller pieces. Therefore, one can assume that there was a rather strong selective pressure for either gene loss, or gene "make-up" to look like other fungal genes. The ecological advantage of getting new abilities for polysaccharide degradation by gene capture may explain that this gene was acquired and made active several times independently. Indeed, acquisition of bacterial GH or other degrading enzymes by fungi has been shown to be advantageous [85].

Our results need now to be generalized by investigating other genes recently transferred from bacteria, in both intron-rich and intron-poor genomes, in order to confirm whether introns colonized intronless genes rapidly, with a density related to the genome average.

## Abbreviations

HGT: Horizontal gene transfer; CBM: Carbohydrate binding module; GH: Glycosyl hydrolase.

## Competing interests

The authors declare no competing interests.

## Authors’ contribution

JLDL designed the study, acquired experimental and database data, analyzed data, and drafted the manuscript. MB brought expertise in Fungi, contributed material and phylogenetic data and analyzed and discussed data. SJ performed GH13 phylogeny, AHV contributed to discussion and manuscript writing, DC performed the datation study and contributed to discussion and manuscript writing. All authors read and approved the final manuscript.

## Supplementary Material

Additional file 1: Table S1 List of primers designed for detection of α-amylase genes orthologous to Phchr1|7087|. PCR conditions were: initial denaturation 94°C, 6 mn; denaturation 94°C, 25 s; annealing 58°C, 50s; elongation 72°C, 1 mn, 45 cycles using the Taq Gold polymerase (Applied Biosystems). Various combinations of forward and reverse primers were tried. Click here for file

Additional file 2: Table S2 GenBank or JGI accession numbers of sequences EF1α, RNA polymerase II LSU 1 and LSU2, used for datation estimates. Click here for file

Additional file 3: Table S3 Abbreviations used in Figure 1, and JGI or Uniprot accession numbers. Colors are as in Figure 1. Click here for file

Additional file 4: Figure S1 Alignment of the α-amylase protein sequences studied, built with MAFFT, showing the intron positions. Pink: phase 0 introns; green: phase 1 introns; blue: phase 2 introns. This alignment was used, without the N-terminal variable region (signal peptide), for gene tree reconstruction (Additional file 5: Figure S2). Intron-slided introns are not shown. Click here for file

Additional file 5: Figure S2 Gene tree drawn from maximum likelihood reconstruction and 100 bootstrap replicates (see text). The tree was rooted with two bacterial sequences. Abbreviations are given in Additional file 2: Table S1. Click here for file

Additional file 6: Figure S3 Reconciliation tree made from the gene tree (Additional file 5: Figure S2) and the species tree (Additional file 7: Figure S4) with Notung 2.6. The letter D indicate gene duplications, grey branches are lost genes. Orange lines are weak edges. Click here for file

Additional file 7: Figure S4 Fungal species tree and divergence times estimated with BEAST using EF1α+LSU1+LSU2, with dates of divergence at nodes. Horizontal bars show the 95% highest posterior intervals of the divergence times. Click here for file

Additional file 8: Table S4 Analysis of exon size distribution. For each gene, the effective number of exon was computed according to ref. 16. The statistical significance was estimated by 10,000 simulations. Ne: effective number of exons. ns: not significant; *: p<0.05. 5%: value of Ne below which are the smallest 5% simulated Ne values; 95%: value of Ne below which are the smallest 5% simulated Ne values. Click here for file

Additional file 9: Figure S5 Consensus sequences at positions -2 and -1, and +1 and +2 around intron positions with different phases, drawn with Weblogo 3.2 [72]. n is the number of sequences. Error bars are as in Figure 6. A: Phase 0 positions, in the presence of intron; B: Phase 1 positions, in the presence of intron; C: Phase 2 positions, in the presence of intron; D: phase 0 positions, in the absence of intron; E: phase 1 positions, in the absence of intron; F: phase 2 positions, in the absence of intron. Click here for file

Additional file 10: Figure S6 Consensus splicing sites of introns drawn with Weblogo 3.2 [72]. Left to the vertical dashed line: first five nucleotides of the 5' splicing site; right to the vertical dashed line: last three nucleotides of the 3' splicing. n is the number of sequences. Error bars are as in Figure 6. A: global consensus; B: conserved old introns; C: recent introns; D: position 30; E: position 56; F: introns of Heterobasidion annosum; G: introns of Punctularia strigosozonata. Click here for file
