# Supplementary material for: Gene make-up: rapid and massive intron gains after horizontal transfer of a bacterial α-amylase gene to Basidiomycetes
Source: BMC Evol Biol. 2013 Feb 13;13:40. doi: 10.1186/1471-2148-13-40 (PMC3584928; doi:10.1186/1471-2148-13-40)
Supplement: Additional file 1: Table S1 — List of primers designed for detection of α-amylase genes orthologous to Phchr1|7087|. PCR conditions were: initial denaturation 94°C, 6 mn; denaturation 94°C, 25 s; annealing 58°C, 50s; elongation 72°C, 1 mn, 45 cycles using the Taq Gold polymerase (Applied Biosystems). Various combinations of forward and reverse primers were tried. [file 1471-2148-13-40-S1.doc]

**Supplementary Table 2**: List of primers designed for detection of α-amylase genes orthologous to Phchr1|7087|. PCR conditions were: initial denaturation 94°C, 6 mn; denaturation 94°C, 25 s; annealing 58°C, 50s; elongation 72°C, 1 mn, 45 cycles using the Taq Gold polymerase (Applied Biosystems). Various combinations of forward and reverse primers were tried.

| Primer name | sequence | position and sense (relative to *P. chrysosporium*) |
| --- | --- | --- |
| PHANDIR1 | CAGATGTTCGARTGGACNTGG | 112-132(+) |
| PHANREV1 | GATRTAGGACACNGGYTGRTA | 241-261(-) |
| PHANDIR2 | TGGTGGACCNNNTAYCARCCNGT | 229-251(+) |
| PHANREV2 | CCTGCNCCRTAGATNACYTCYTG | 873-895(-) |
| PHANDIR3D | TTCGTCGAYAAYCAYGAYAC | 1206-1225(+) |
| PHANDIR3T | TTCGTCACNAAYCAYGAYAC | 1206-1225(+) |
| PHANREV3 | CCGCGNCCGAANGCDATYTG | 1625-1644(-) |
