# Supplementary material for: Gene make-up: rapid and massive intron gains after horizontal transfer of a bacterial α-amylase gene to Basidiomycetes
Source: BMC Evol Biol. 2013 Feb 13;13:40. doi: 10.1186/1471-2148-13-40 (PMC3584928; doi:10.1186/1471-2148-13-40)
Supplement: Additional file 2: Table S2 — GenBank or JGI accession numbers of sequences EF1α, RNA polymerase II LSU 1 and LSU2, used for datation estimates. [file 1471-2148-13-40-S2.doc]

**Supplementary Table 1 :** abbreviations used in Figure 1, and JGI or Uniprot accession numbers. Colors are as in Figure 1.

| GH13  subfamily | Source | Abbreviation | UniProt or JGI identifier | Taxonomy |
| --- | --- | --- | --- | --- |
|  | *Phanerochaete chrysosporium* RP78 | Phchr7087 | jgi|Phchr1|7087| | Basidiomycetes Agaricomycetes |
|  | *Phanerochaete carnosa* HHB-10118-Sp | Phcar259593 | jgi|Phaca1|259593| | Basidiomycetes Agaricomycetes |
|  | *Phanerochaete carnosa* HHB-10118-Sp | Phcar259591 | jgi|Phaca1|259591| | Basidiomycetes Agaricomycetes |
|  | *Phlebiopsis gigantea* | Phlgi33454 | jgi|Phlgi1|33454| | Basidiomycetes Agaricomycetes |
|  | *Ceriporiopsis subvermispora* B | Cersu93449 | jgi|Cersu1|93449| | Basidiomycetes Agaricomycetes |
|  | *Ceriporiopsis subvermispora* B | Cersu156021 | jgi|Cersu1|156021| | Basidiomycetes Agaricomycetes |
|  | *Phlebia brevispora* | Phlbr17677 | jgi|Phlbr1|17677| | Basidiomycetes Agaricomycetes |
|  | *Ganoderma sp.* 10597 SS1 | Gansp123688 | jgi|Gansp1|123688| | Basidiomycetes Agaricomycetes |
|  | *Ganoderma sp.* 10597 SS1 | Gansp105279 | jgi|Gansp1|105279| | Basidiomycetes Agaricomycetes |
|  | *Trametes versicolor* | Trave30524 | jgi|Trave1|30524| | Basidiomycetes Agaricomycetes |
|  | *Dichomitus squalens* | Dicsq125666 | jgi|Dicsq1|125666| | Basidiomycetes Agaricomycetes |
|  | *Dichomitus squalens* | Dicsq68009 | jgi|Dicsq1|68009| | Basidiomycetes Agaricomycetes |
|  | *Agaricus bisporus var bisporus* (H97) | Agabi211295 | jgi|Agabi_varbisH97_2|211295| | Basidiomycetes Agaricomycetes |
|  | *Schizophyllum commune* | Schco67047 | D8Q2K1 | Basidiomycetes Agaricomycetes |
|  | *Schizophyllum commune* | Schco107514 | D8Q2K0 | Basidiomycetes Agaricomycetes |
|  | *Pleurotus ostreatus* PC15 | PleosPC15_1095839 | jgi|PleosPC15_2|1095839| | Basidiomycetes Agaricomycetes |
|  | *Pleurotus ostreatus* PC15 | PleosPC15_20823 | jgi|PleosPC15_2|20823| | Basidiomycetes Agaricomycetes |
|  | *Pleurotus ostreatus* PC9 | PleosPC9_91049 | jgi|PleosPC9_1|91049| | Basidiomycetes Agaricomycetes |
|  | *Coniophora puteana* | Conpu108744 | jgi|Conpu1|108744| | Basidiomycetes Agaricomycetes |
|  | *Serpula lacrymans* S7_9 | SerlaS7_9_416486 | F8Q2E0 | Basidiomycetes Agaricomycetes |
|  | *Stereum hirsutum* FP-91666 SS1 | Stehi83072 | jgi|Stehi1|83072| | Basidiomycetes Agaricomycetes |
|  | *Stereum hirsutum* FP-91666 SS1 | Stehi159685 | jgi|Stehi1|159685| | Basidiomycetes Agaricomycetes |
|  | *Stereum hirsutum* FP-91666 SS1 | Stehi78757 | jgi|Stehi1|78757| | Basidiomycetes Agaricomycetes |
|  | *Stereum hirsutum* FP-91666 SS1 | Stehi95395 | jgi|Stehi1|95395| | Basidiomycetes Agaricomycetes |
|  | *Heterobasidion annosum* | Hetan65781 | jgi|Hetan2|65781| | Basidiomycetes Agaricomycetes |
|  | *Punctularia strigosozonata* | Punst74571 | jgi|Punst1|74571| | Basidiomycetes Agaricomycetes |
|  | *Punctularia strigosozonata* | Punst140991 | jgi|Punst1|140991| | Basidiomycetes Agaricomycetes |
|  | *Punctularia strigosozonata* | Punst118997 | jgi|Punst1|118997| | Basidiomycetes Agaricomycetes |
|  | *Gloeophyllum trabeum* | Glotr121909 | jgi|Glotr1_1|121909| | Basidiomycetes Agaricomycetes |
|  | *Gloeophyllum trabeum* | Glotr185585 | jgi|Glotr1_1|185585| | Basidiomycetes Agaricomycetes |
|  | *Fomitiporia mediterranea* | Fomme130910 | jgi|Fomme1|130910| | Basidiomycetes Agaricomycetes |
|  | *Auricularia delicata* SS-5 | Aurde116714 | jgi|Aurde1|116714| | Basidiomycetes Agaricomycetes |
|  | *Dacryopinax sp.* DJM731 SSP1 | Dacsp51007 | jgi|Dacsp1|51007| | Basidiomycetes Agaricomycetes |
|  | *Piriformospora indica* | Pirin_G4TCP6 | G4TCP6 | Basidiomycetes Agaricomycetes |
|  | *Bjerkandera adusta* | Bjead45153 | jgi|Bjead1|45153| | Basidiomycetes Agaricomycetes |
|  | *Bjerkandera adusta* | Bjead55696 | jgi|Bjead1|55696| | Basidiomycetes Agaricomycetes |
|  | *Bjerkandera adusta* | Bjead141648 | jgi|Bjead1|41648| | Basidiomycetes Agaricomycetes |
|  | *Puccinia graminis* | Pucgr25736 | jgi|Pucgr1|25736|, E3K735 | Basidiomycetes Tremellomycete |
|  | *Melampsora larici-populina* | Melpl90587 | jgi|Melpl1|90587|, F4RXF9 | Basidiomycetes Tremellomycete |
| GH13_1 | Agaricus bisporus | Agabi-1 | jgi|Agabi_varbisH97_2|134077| | Basidiomycetes Agaricomycetes |
|  | *Gloeophyllum trabeum* | Glotr-1 | jgi|Glotr1_1|73036| | Basidiomycetes Agaricomycetes |
|  | *Phanerochaete chrysosporium* | Phach-1 | jgi|Phchr1|38357| | Basidiomycetes Agaricomycetes |
|  | *Serpula lacrymans* | Serla-1 | jgi|SerlaS7_3_2|115128| | Basidiomycetes Agaricomycetes |
|  | *Aspergillus oryzae* | Aspor-1 P0C1B3 | P0C1B3 | Ascomycetes Eurotiomycetes |
|  | *Saccharomycopsis fibuligera* | Sacfi-1 D4P4Y7 | D4P4Y7 | Ascomycetes Saccharomycetes |
| GH13_5 | *Histoplasma capsulatum* | Hisca-5 A0T074 | A0T074 | Ascomycetes Eurotiomycetes |
|  | *Paracoccidioides brasiliensis* | Parbr-5 A7L832 | A7L832 | Ascomycetes Eurotiomycetes |
|  | *Bacillus amyloliquefaciens* | Bacam-5 P00692 | P00692 | Firmicutes Bacillales |
|  | *Cytophaga* sp. | Cytsp-5 Q9RQT8 | Q9RQT8 | Bacteroidetes |
|  | ***Methanosalsum zhilinae*** | Meszh-5 F7XP95 | F7XP95 | Euryarcheotes Methanosarcinales |
| GH13_6 | *Hordeum vulgare* | Horvu-6 P00693 | P00693 | Viridiplantae monocot |
|  | *Malus domestica* | Maldo-6 Q5BLY0 | Q5BLY0 | Viridiplantae dicot |
| GH13_7 | *Pyrococcus woesei* | Pyrwo-7 Q7LYT7 | Q7LYT7 | Euryarcheotes Thermococcales |
|  | *Thermococcus hydrothermalis* | Thehy-7 O93647 | O93647 | Euryarcheotes Thermococcales |
| GH13_15 | *Drosophila melanogaster* | Drome-15 P08144 | P08144 | Metazoa Arthropoda |
|  | *Tenebrio molitor* | Tenmo-15 P56634 | P56634 | Metazoa Arthropoda |
| GH13_19 | *Bacillus halodurans* | Bacha-19 A8QWV3 | A8QWV3 | Firmicutes Bacillales |
|  | *Escherichia coli* | Escco-19 P25718 | P25718 | γ-Proteobacteria |
| GH13_24 | *Gallus gallus* | Galga-24 Q98942 | Q98942 | Metazoa Vertebrata |
|  | *Homo sapiens* | Homsa-24 P04746 | P04746 | Metazoa Vertebrata |
| GH13_27 | *Aeromonas hydrophila* | Aerhy-27 P22630 | P22630 | Firmicutes Bacillales |
|  | *Xanthomonas campestris* | Xanca-27 Q56791 | Q56791 | γ-Proteobacteria |
| GH13_28 | *Bacillus subtilis* | Bacsu-28 P00691 | P00691 | Firmicutes Bacillales |
|  | *Lactobacillus amylovorus* | Lacam-28 Q48502 | Q48502 | Firmicutes Lactobacillales |
| GH13_32 | *Kocuria varians* | Kocva-32 E5RKQ5 | E5RKQ5 | Actinobacteria Actinomycetales |
|  | *Streptomyces limosus* | Strli-32 P09794 | P09794 | Actinobacteria Actinomycetales |
|  | *Streptomyces venezuelae* | Strve-32 P22998 | P22998 | Actinobacteria Actinomycetales |
|  | *Thermomonospora curvata* | Thscu-32 P29750 | P29750 | Actinobacteria Actinomycetales |
|  | *Salinispora tropica* | Saltr-32 A4X6F5 | A4X6F5 | Actinobacteria Actinomycetales |
|  | *Herpetosiphon aurantiacus* | Herau-32 A9AVU7 | A9AVU7 | Chloroflexi herpetosiphonales |
| GH13_? | *Pseudoalteromonas halaoplanktis* | Psaha-? P29957 | P29957 | γ-Proteobacteria |
| GH13_36 | *Anaerobranca gottschalkii* | Anago-36 Q5I942 | Q5I942 | Firmicutes Clostridia |
|  | *Halothermothrix orenii* | Hator-36 Q8GPL8 | Q8GPL8 | Firmicutes Clostridia |
