# Supplementary material for: Gene make-up: rapid and massive intron gains after horizontal transfer of a bacterial α-amylase gene to Basidiomycetes
Source: BMC Evol Biol. 2013 Feb 13;13:40. doi: 10.1186/1471-2148-13-40 (PMC3584928; doi:10.1186/1471-2148-13-40)
Supplement: Additional file 3: Table S3 — Abbreviations used in Figure 1, and JGI or Uniprot accession numbers. Colors are as in Figure 1. [file 1471-2148-13-40-S3.doc]

**Supplementary Table 3:** GenBank or JGI accession numbers of sequences EF1α, RNA polymerase II LSU 1 and LSU2, used for datation estimates

| **species** | **EF1-alpha** | **RNApol II LSU1** | **RNApol II LSU2** |
| --- | --- | --- | --- |
| *Agaricus_bisporus* | Agabi_varbisH97_2|133083| | Agabi_varbisH97_2|215454| | Agabi_varbisH97_2|113824| |
| *Aspergillus_clavatus* | XP_001269546 | XP_001268791 | XP_001272355 |
| *Aspergillus_flavus* | XP_002380898 | XP_002374837 | XP_002380667 |
| *Aspergillus_fumigatus* | XP_750388 | XP_752837 | XP_746740 |
| *Aspergillus_niger* | XP_001398942 | XP_001389676 | XP_001395161 |
| *Aspergillus_oryzae* | XP_001823843 | BAB12227 | XP_001825469 |
| *Aspergillus_terreus* | XP_001212188 | XP_001210766 | XP_001209185 |
| *Auricularia_delicata* | Aurde1|109924| | Aurde1|50641| | Aurde1|111514| |
| *Bjerkandera_adusta* | Bjead1_1|33809| | Bjead1_1|45271| | Bjead1_1|163844| |
| *Botryobasidium_botryosum* | Botbo1|34109| | Botbo1|29699| | Botbo1|27165| |
| *Candida_glabrata* | XP_445466 | XP_447415 | XP_448959 |
| *Ceriporiopsis_subvermispora* | Cersu1|42781| | Cersu1|110068| | Cersu1|110450| |
| *Chaetomium_globosum* | XP_001222639 | XP_001220925 | XP_001226434 |
| *Coccidioides_immitis* | AAK54650 | XP_001243803 | XP_001240650 |
| *Coniophora_puteana* | Conpu1|78944| | Conpu1|148321| | Conpu1|116067| |
| *Coprinopsis_cinerea* | XP_001828758 | Copci1|13692| | XP_001829140 |
| *Cryptococcus_neoformans* | XP_568462 | Cryne_H99_1|1954| | XP_775871 |
| *Dacryopinax_sp* | Dacsp1|22324| | Dacsp1|118311| | Dacsp1|23500| |
| *Dichomitus_squalens* | Dicsq1|46769| | Dicsq1|66181| | Dicsq1|164685| |
| *Fomitiporia_mediterranea* | Fomme1|140043 | Fomme1|167708| | Fomme1|19006| |
| *Fomitopsis_pinicola* | Fompi1|159202| | Fompi1|152872| | AAV53362 |
| *Fusarium_oxysporum* | EGU8320 | FOXG_00887.2 | EGU88188 |
| *Ganoderma_sp* | Gansp1|113496| | Gansp1|113051| | Gansp1|148067| |
| *Gib_moniliformis* | AEH68817 | XP388987 | AAS80336 |
| *Gibberella_zeae* | XP_388987 | XP_381092 | ADR31307 |
| *Gloeophyllum_trabeum* | Glotr1_1|109132| | Glotr1_1|30111| | Glotr1_1|135096| |
| *Heterobasidion_annosum* | Hetan2|406970| | Hetan2|424453| | Hetan2|443043| |
| *Jaapia_argillacea* | Jaaar1|29726| | Jaaar1|197811| | Jaaar1|121085| |
| *Kluyveromyces_lactis* | XP_451929 | XP_455310 | XP_451784 |
| *Laccaria_bicolor* | XP_001873214 | Lacbi2|690618| | Lacbi1|243796| |
| *Magnaporthe_grisea* | XP_361098 | MGG_04652.6 | XP_362269 |
| *Nectria_haematococca* | EEU47496 | XP_003054466 | EEU39607 |
| *Neosartorya_fischeri* | XP_001265028 | XP_001264289 | XP_001262829 |
| *Neurospora_crassa* | XP_964868 | XP_964097 | CAD70445 |
| *Neurospora_discreta* | [Neudi1|160296|](http://genome.jgi-psf.org/cgi-bin/dispGeneModel?db=Neudi1&id=160296) | Neudi1|73118| | [Neudi1|128789|](http://genome.jgi-psf.org/cgi-bin/dispGeneModel?db=Neudi1&id=128789) |
| *Neurospora_tetrasperma* | [EGO51750](http://genome.jgi-psf.org/cgi-bin/dispGeneModel?db=Neute_matA2&id=118521) | EGO51638 | EGO54804 |
| *Phanerochaete carnosa* | Phaca1|168368| | Phaca1|246551| | Phaca1|247866| |
| *Phanerochaete_chrysosporium* | Phchr1|134660| | [Phchr1|127142|](http://genome.jgi-psf.org/cgi-bin/dispGeneModel?db=Phchr1&id=127142) | Phchr1|7545| |
| *Phlebia_brevispora* | Phlbr1|26354| | Phlbr1|28429| | Phlbr1|26149| |
| *Pichia_stipitis* | XP_001382687 | Picst3|75374| | XP_001387366 |
| *Piriforma_indica* | CCA69108 | CCA67728 | CCA75371 |
| *Pleurotus_ostreatus* | PleosPC15_2|1070256| | PleosPC15_2|24928| | PleosPC15_2|1062993| |
| *Podospora_anserina* | XP_001907437 | XP_001912461 | XP_001903788 |
| *Punctularia_strigosozonata* | Punst1|41184| | Punst1|123387| | Punst1|48856| |
| *Schizophyllum_commune* | Schco1|84142| | Schco1|81059| | Schco1|73589| |
| *Serpula lacrymans* | SerlaS7_3_2|175951| | SerlaS7_3_2|98944| | SerlaS7_3_2|101102| |
| *Sporotrichum_thermophile* | AEO54928 | Spoth2|2294525| | AEO59342 |
| *Stereum_hirsutum* | Stehi1|88829| | Stehi1|46012| | Stehi1|89162| |
| *Thielavia_terrestris* | AEO65053 | Thite2|2121340| | AEO64668 |
| *Trametes_versicolor* | AAY46269 | Trave1|110086| | ABD65895 |
| *Trichoderma_atroviride* | ACO37562 | Triat2|89498| | [jgi|Triat2|151043|](http://genome.jgi-psf.org/cgi-bin/dispGeneModel?db=Triat2&id=151043) |
| *Trichoderma_reesei* | EGR49982 | Trire2|79315| | EGR47390 |
| *Trichoderma_virens* | Trive1|83874| | TriviGv29_8_2|85034| | Trive1|76818| |
| *Verticillium_dahliae* | EGY21035 | VDAG_06114.1 | EGY14461 |
| *Wolfiporia_cocos* | Wolco1|63885| | Wolco1|134958| | Wolco1|135432| |
