# Supplementary figures and images for: Gene make-up: rapid and massive intron gains after horizontal transfer of a bacterial α-amylase gene to Basidiomycetes
Source: BMC Evol Biol. 2013 Feb 13;13:40. doi: 10.1186/1471-2148-13-40 (PMC3584928; doi:10.1186/1471-2148-13-40)

## Slide 1
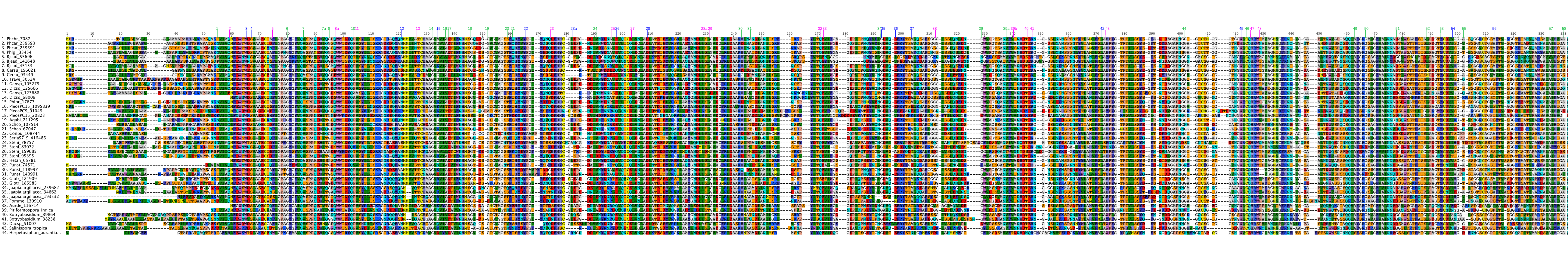

1
2
3
4
5
6
7
7a
8
8a
10
11
12
13
14
15
16
17
18
19
20
21
22
23
23a
24
25
26
27
28
28a
29
30
31
32
33
34
35
36
37
38
39
39a
39b
40
41
42
43
44
45
46
47
48
49
50
51
52
53
54
55
56
57
58

Supplement: Additional file 4: Figure S1 — Alignment of the α-amylase protein sequences studied, built with MAFFT, showing the intron positions. Pink: phase 0 introns; green: phase 1 introns; blue: phase 2 introns. This alignment was used, without the N-terminal variable region (signal peptide), for gene tree reconstruction (Additional file 5: Figure S2). Intron-slided introns are not shown. [file 1471-2148-13-40-S4.ppt]

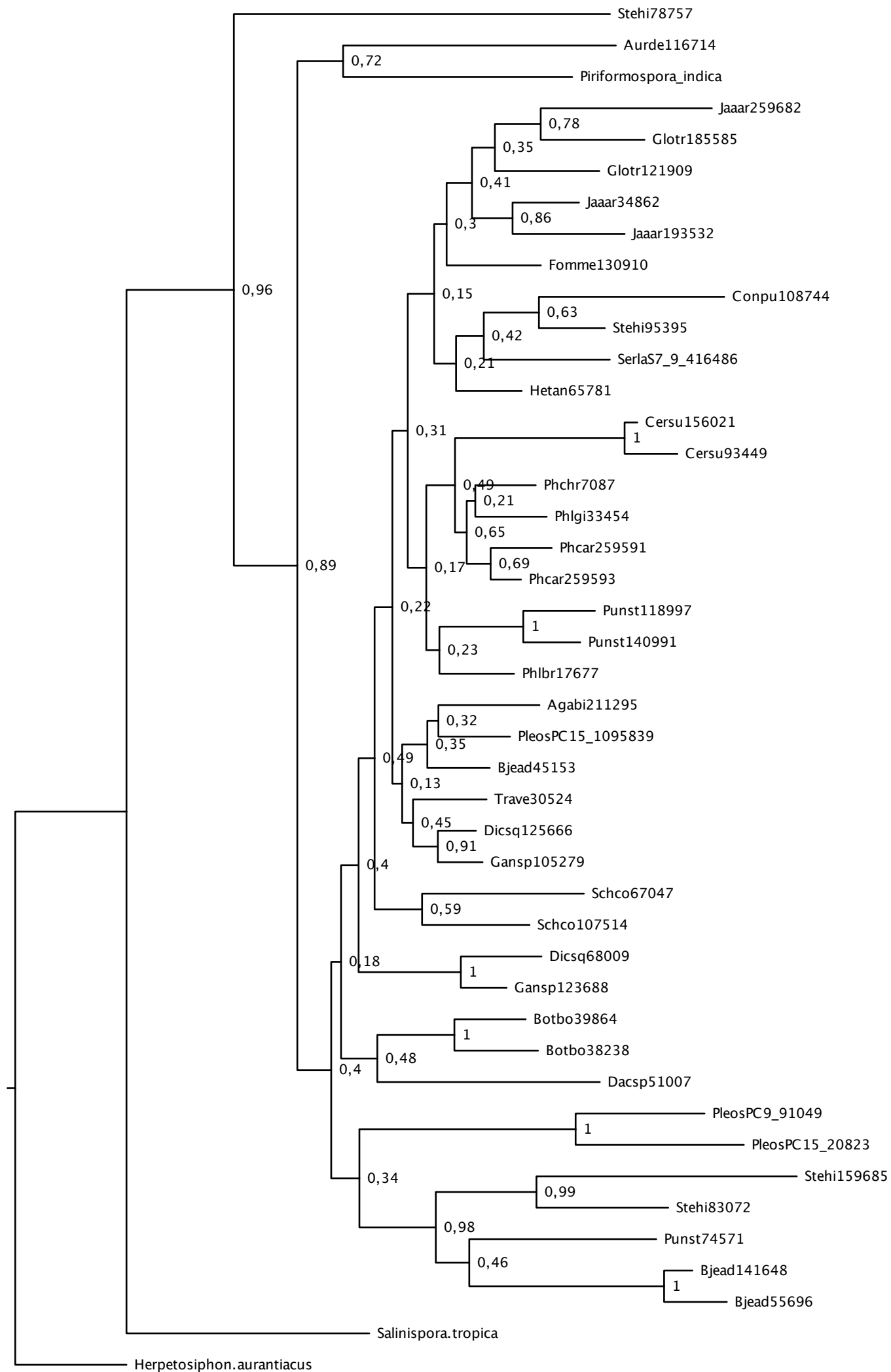

0.2

Supplement: Additional file 5: Figure S2 — Gene tree drawn from maximum likelihood reconstruction and 100 bootstrap replicates (see text). The tree was rooted with two bacterial sequences. Abbreviations are given in Additional file 2: Table S1. [file 1471-2148-13-40-S5.pdf]

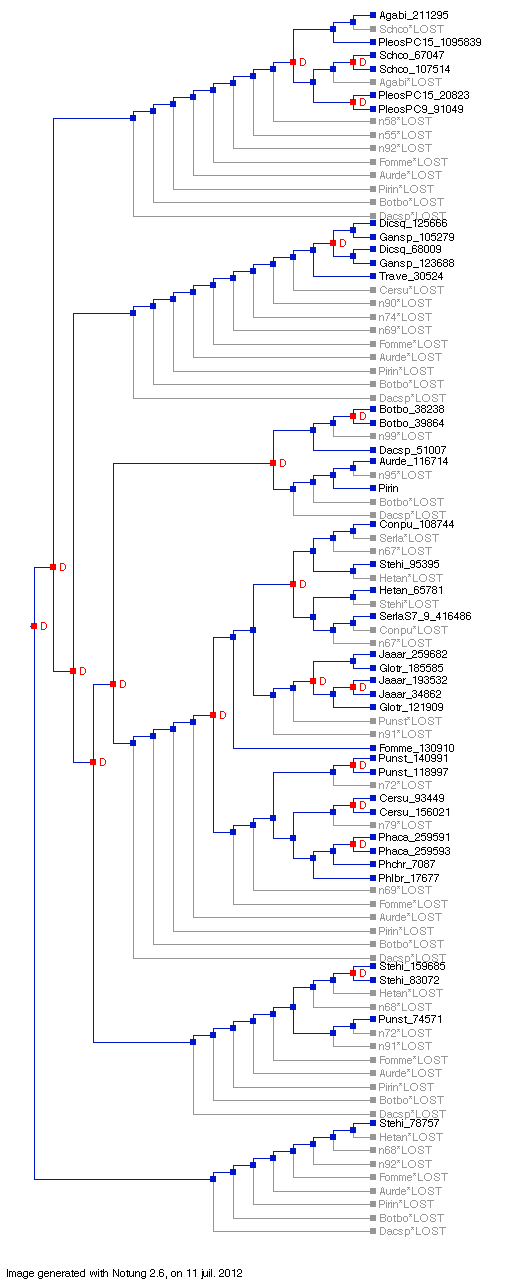

Supplement: Additional file 6: Figure S3 — Reconciliation tree made from the gene tree (Additional file 5: Figure S2) and the species tree (Additional file 7: Figure S4) with Notung 2.6. The letter D indicate gene duplications, grey branches are lost genes. Orange lines are weak edges. [file 1471-2148-13-40-S6.png]

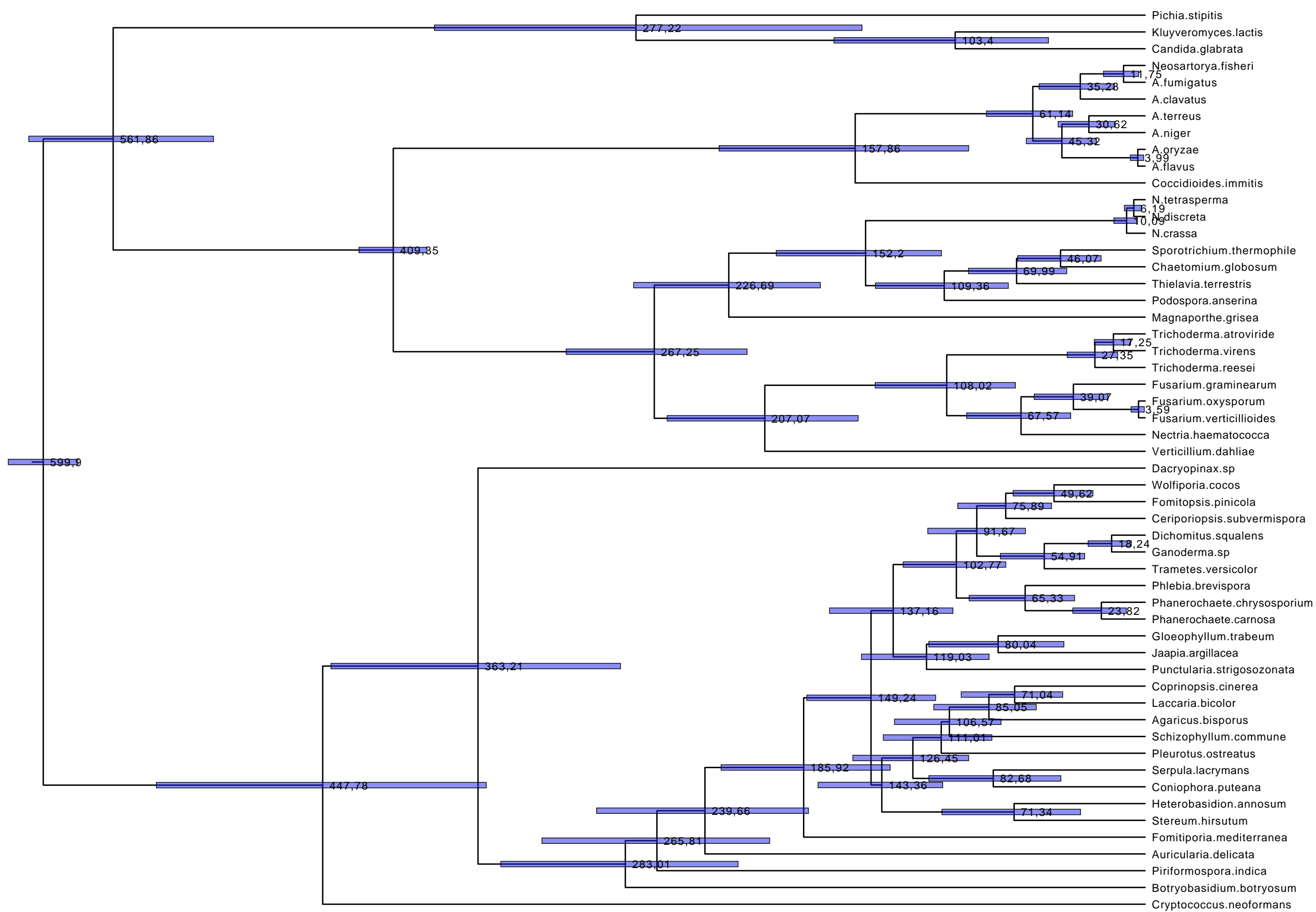

60.0

Supplement: Additional file 7: Figure S4 — Fungal species tree and divergence times estimated with BEAST using EF1α+LSU1+LSU2, with dates of divergence at nodes. Horizontal bars show the 95% highest posterior intervals of the divergence times. [file 1471-2148-13-40-S7.pdf]

## Slide 1
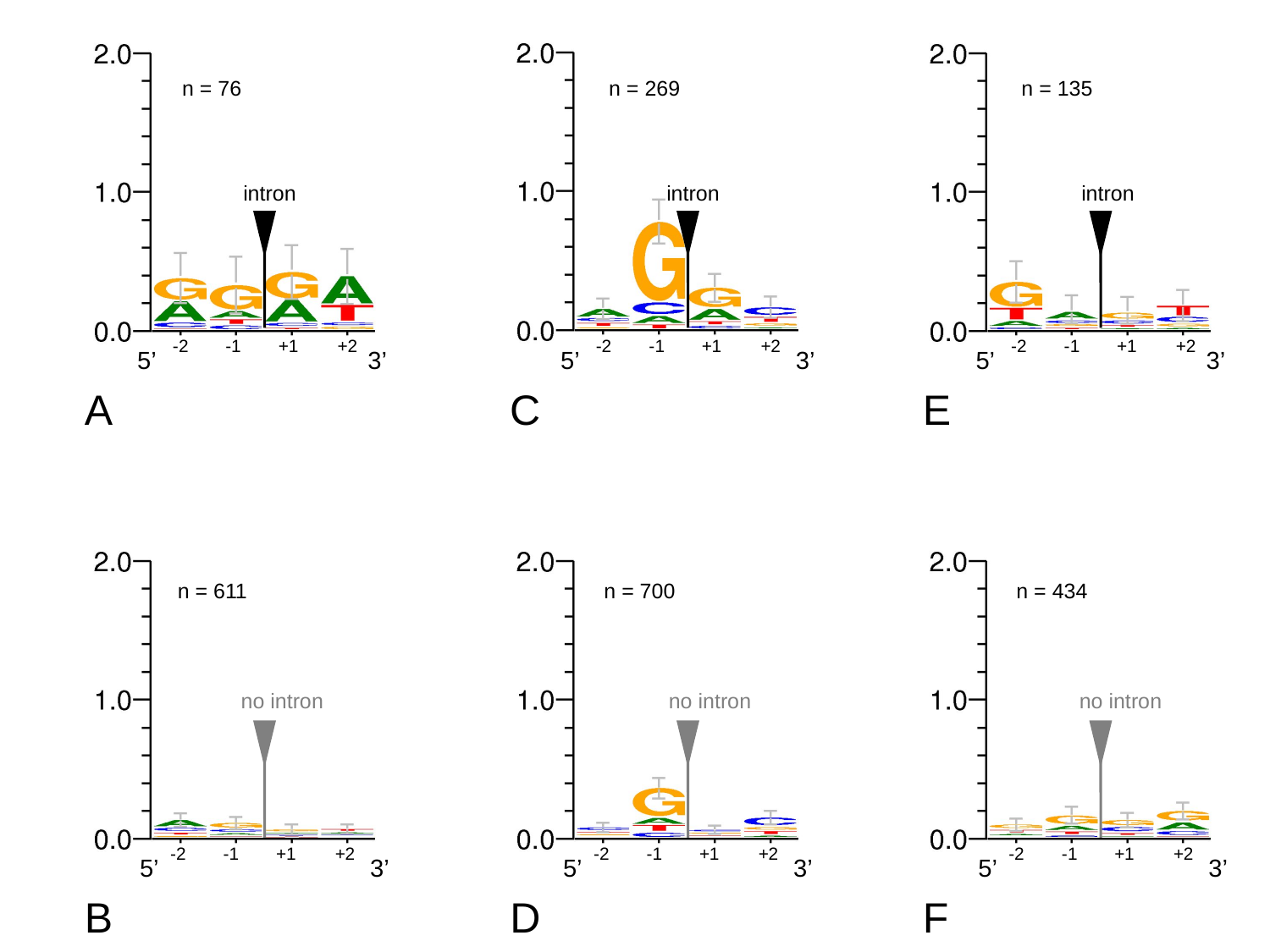

n = 76
n = 269
n = 135
intron
intron
intron
-2
-1
+1
+2
-2
-1
+1
+2
-2
-1
+1
+2
5’
3’
5’
3’
5’
3’
A
C
E
n = 611
n = 700
n = 434
no intron
no intron
no intron
-2
-1
+1
+2
-2
-1
+1
+2
-2
-1
+1
+2
5’
3’
5’
3’
5’
3’
B
D
F

Supplement: Additional file 9: Figure S5 — Consensus sequences at positions -2 and -1, and +1 and +2 around intron positions with different phases, drawn with Weblogo 3.2 [72]. n is the number of sequences. Error bars are as in Figure 6. A: Phase 0 positions, in the presence of intron; B: Phase 1 positions, in the presence of intron; C: Phase 2 positions, in the presence of intron; D: phase 0 positions, in the absence of intron; E: phase 1 positions, in the absence of intron; F: phase 2 positions, in the absence of intron. [file 1471-2148-13-40-S9.ppt]

## Slide 1
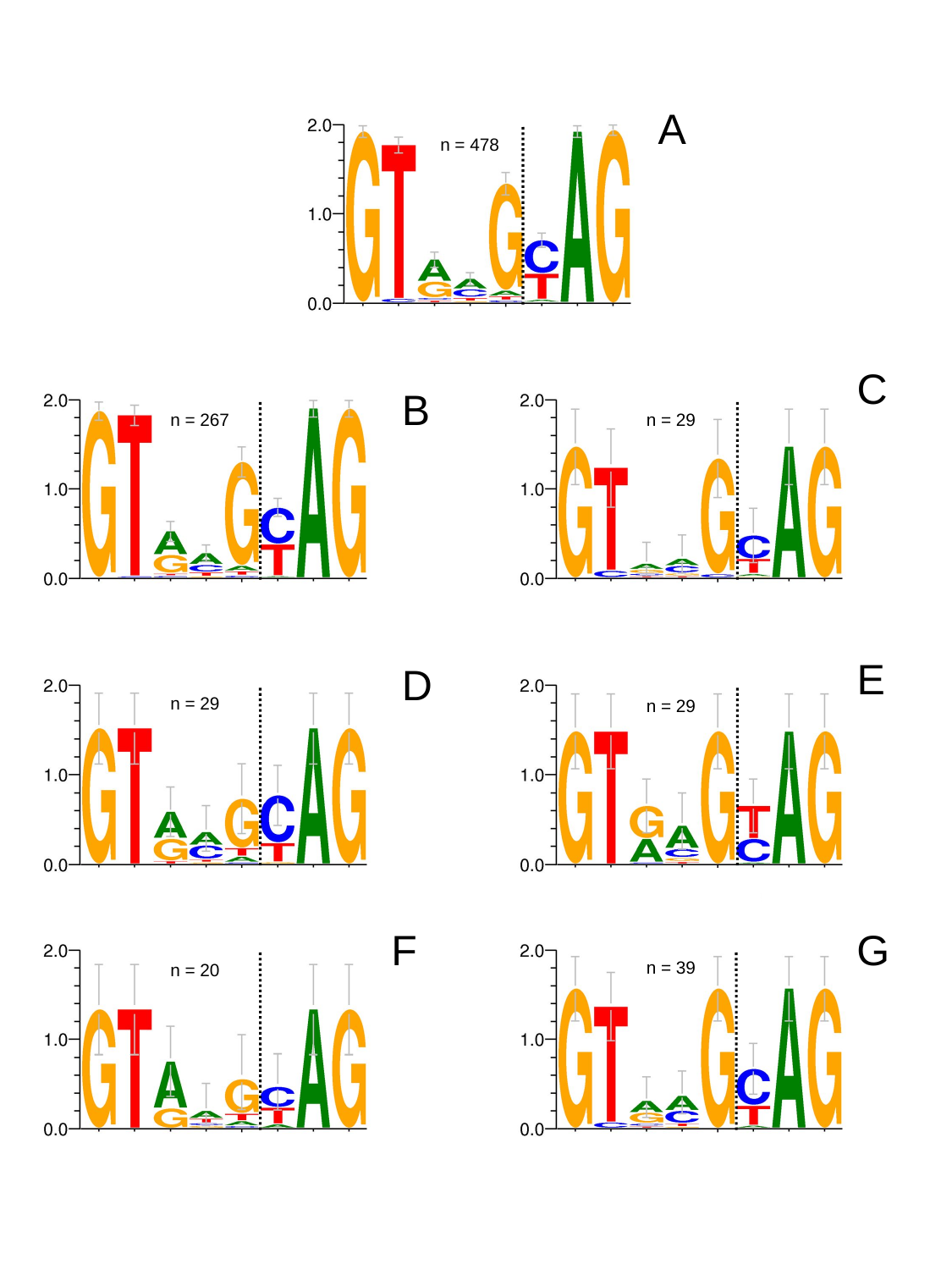

A
n = 478
C
B
n = 267
n = 29
E
D
n = 29
n = 29
F
G
n = 39
n = 20

Supplement: Additional file 10: Figure S6 — Consensus splicing sites of introns drawn with Weblogo 3.2 [72]. Left to the vertical dashed line: first five nucleotides of the 5' splicing site; right to the vertical dashed line: last three nucleotides of the 3' splicing. n is the number of sequences. Error bars are as in Figure 6. A: global consensus; B: conserved old introns; C: recent introns; D: position 30; E: position 56; F: introns of Heterobasidion annosum; G: introns of Punctularia strigosozonata. [file 1471-2148-13-40-S10.ppt]
